# Novel hub genes and regulatory network related to ferroptosis in tetralogy of Fallot

**DOI:** 10.3389/fped.2023.1177993

**Published:** 2023-10-18

**Authors:** Yu Wang, Junjie Yang, Jieru Lu, Qingjie Wang, Jian Wang, Jianyuan Zhao, Yuqiang Huang, Kun Sun

**Affiliations:** ^1^ Department of Pediatric Cardiology, The Second Affiliated Hospital, Yuying Children’s Hospital of Wenzhou Medical University, Wenzhou, China; ^2^ Department of Pediatric Cardiology, Xinhua Hospital, School of Medicine, Shanghai Jiao Tong University, Shanghai, China; ^3^ Linyi Maternal and Child Health Care Hospital, Linyi, China

**Keywords:** TOF, ferroptosis, GEO, DEmRNAs, DEmiRs, network

## Abstract

Ferroptosis is a newly discovered type of cell death mainly triggered by uncontrolled lipid peroxidation, and it could potentially have a significant impact on the development and progression of tetralogy of Fallot (TOF). Our project aims to identify and validate potential genes related to ferroptosis in TOF. We obtained sequencing data of TOF from the GEO database and ferroptosis-related genes from the ferroptosis database. We employed bioinformatics methods to analyze the differentially expressed mRNAs (DEmRNAs) and microRNAs between the normal control group and TOF group and identify DEmRNAs related to ferroptosis. Protein–protein interaction analysis was conducted to screen hub genes. Furthermore, a miRNA–mRNA–TF co-regulatory network was constructed to utilize prediction software. The expression of hub genes was further validated through quantitative real-time reverse-transcription polymerase chain reaction (qRT-PCR). After conducting the differential gene analysis, we observed that in TOF, 41 upregulated mRNAs and three downregulated mRNAs associated with ferroptosis genes were found. Further Gene Ontology/Kyoto Encyclopedia of Genes and Genomes analysis revealed that these genes were primarily involved in molecular functions and biological processes related to chemical stress, oxidative stress, cellular response to starvation, response to nutrient levels, cellular response to external stimulus, and cellular response to extracellular stimulus. Furthermore, we constructed a miRNA–mRNA–TF co-regulatory network. qRT-PCR analysis of the right ventricular tissues from human cases showed an upregulation in the mRNA levels of KEAP1 and SQSTM1. Our bioinformatics analysis successfully identified 44 potential genes that are associated with ferroptosis in TOF. This finding significantly contributes to our understanding of the molecular mechanisms underlying the development of TOF. Moreover, these findings have the potential to open new avenues for the development of innovative therapeutic approaches for the treatment of this condition.

## Introduction

Congenital heart disease (CHD) is a structural abnormality of the heart and large blood vessels that occurs during embryonic development, resulting in the persistence of holes or channels that should normally close after birth. It is the most common birth defect and a major cause of infant mortality. Epidemiological studies indicate that the incidence of congenital heart disease is around 1%, with tetralogy of Fallot (TOF) accounting for 5%–10% of all cases, affecting approximately three in every 10,000 live births (1). TOF is characterized by four typical features, namely, ventricular septal defect, overriding aorta, pulmonary artery stenosis, and right ventricular hypertrophy (2). This condition is classified as a conotruncal defect and is believed to result from incomplete separation between the truncus arteriosus and the bulbus arteriosus during early embryonic development (3). However, the precise molecular mechanisms underlying the pathogenesis of TOF remain not fully understood.

Recently, a novel form of iron-dependent non-apoptotic regulated cell death known as ferroptosis has emerged as a significant player in various biological processes. The hallmark features of ferroptosis include an unbalanced iron steady-state and lipid peroxidation resulting from the accumulation of excessive reactive oxygen species (ROS) (4). Emerging evidence suggests that ferroptosis plays a crucial role in heart development and pathology (5). For instance, extensive ferroptosis has been observed in placental trophoblasts associated with conditions such as preeclampsia or fetal growth restriction, both within and outside the villi. This indicates that iron-dependent cell death is essential for healthy pregnancy. Aberrant ferroptosis can lead to increased trophoblast death, disrupting the regular turnover of extra villous cytotrophoblasts and hampering the normal recruitment of syncytiotrophoblasts (6). Studies have also demonstrated the involvement of ferroptosis in the pathogenesis of TOF. *In vitro* and *in vivo* experiments using micro-222 knock-in mice offspring have shown that ferroptosis is a critical molecular mechanism underlying the development of TOF (7). Offspring of mothers with type 2 diabetes also exhibit outflow tract (OFT) abnormalities, which can be attributed to oxidative stress and ferroptosis (8, 9). Furthermore, experiments conducted on H9C2 cells have revealed that induction of ferroptosis in cardiac myocytes leads to significant downregulation of TBX1 and miR-193a-3p expression, while TGF-β2 expression is upregulated in the human embryonic CHD tissue. These findings suggest that ferroptosis may underlie the molecular mechanisms associated with TOF (10). In summary, the discovery of ferroptosis as an iron-dependent regulated cell death pathway has shed light on its crucial role in heart development and disease. Understanding the intricate molecular mechanisms underlying ferroptosis may provide novel insights into the pathogenesis of congenital heart defects such as TOF.

In this study, we aimed to integrate ferroptosis with TOF by utilizing the Gene Expression Omnibus (GEO) database. Through a comprehensive analysis, we identified hub genes that may play a crucial role in the pathogenesis of TOF and their association with ferroptosis. In addition, we constructed an integrative regulatory network involving miRNAs, mRNAs, and transcription factors (TFs) to gain further insights into the underlying mechanisms of TOF. By identifying these hub genes, we have potentially discovered new diagnostic biomarkers that are closely related to the occurrence and progression of ferroptosis in TOF. Further investigation into the functions and regulatory pathways of these hub genes may provide valuable information for the development of diagnostic tools and therapeutic strategies for TOF.

## Materials and methods

### Data acquisition

We obtained gene expression datasets from the GEO database to investigate the molecular changes associated with TOF. Specifically, data expression datasets GSE35776 (platform: GPL5175) (11), GSE26125 (platform: GPL11329) (12), GSE35490 (platform: GPL8786) (11), and GSE40128 (platform: GPL8786) (13) were downloaded (14, 15). The datasets GSE35776 and GSE35490 each contain eight samples from healthy infants and 16 samples from pediatric TOF patients. The dataset GSE26125 contains five samples from healthy infants and 16 samples from pediatric TOF patients. The dataset GSE40128 contains three samples from healthy infants and five samples from pediatric TOF patients.

### Identification of differentially expressed mRNAs and miRNAs

Differential expression analysis of mRNAs and miRNAs between the TOF and control groups was performed using the GEO2R tool. In our analysis, we considered mRNAs and miRNAs with an *adjusted p-value* of <0.05 and a |*log fold change (FC)*| of >1 as differentially expressed (16).

To investigate the potential involvement of ferroptosis in TOF, we obtained a dataset comprising 259 ferroptosis-related genes from a ferroptosis database (17). We then compared this dataset with the list of differentially expressed genes obtained from GSE35776 and GSE26125 datasets to identify the differentially expressed mRNAs (DEmRNAs) associated with ferroptosis in TOF. To visualize the overlapping genes, Venn diagram analysis was performed using the OmicStudio tools available at https://www.omicstudio.cn/tool.

### Gene Ontology and Kyoto Encyclopedia of Genes and Genomes pathway enrichment analysis

To gain insights into the potential biological functions and underlying mechanisms of the ferroptosis-associated genes identified in TOF, we performed Gene Ontology (GO) and Kyoto Encyclopedia of Genes and Genomes (KEGG) pathway enrichment analysis. For this purpose, we utilized R packages such as “clusterProfiler,” “enrichplot,” and “ggnewscale.” The “clusterProfiler” package allowed us to perform enrichment analysis by mapping the target genes to GO terms and KEGG pathways. Subsequently, the “enrichplot” package was employed to generate visualizations, such as enriched GO term and KEGG pathway plots. In addition, the “ggnewscale” package facilitated the customization of color scales in the generated plots, enhancing their interpretability (18).

### PPI network construction and identification of hub genes

To explore the interactions among the differentially expressed ferroptosis-related mRNAs (DEmRNAs), we utilized the STRING online database (https://string-db.org/) (19). By setting an interaction score of 0.4 as the cut-off, we identified the potential interactions between these DEmRNAs. The STRING database provides a comprehensive view of protein–protein interactions (PPI) and functional associations.

To visualize the network of interactions, we used Cytoscape software (version 3.9.1) (20). In the network diagram, each gene was represented by a node, and the interactions between genes were represented by edges. We assigned different colors to represent upregulated genes (red) and downregulated genes (green). This visualization allowed us to gain a visual understanding of the interconnectedness among the ferroptosis-related DEmRNAs in TOF. For gene network cluster analysis and identification of important hub genes, we utilized the CytoNCA application available in Cytoscape. CytoNCA employs various network centrality measures to identify key genes within the network. This analysis helped us determine the hub genes, which play crucial roles in the regulation and functioning of the gene network associated with ferroptosis in TOF (21).

### miRNA–target regulatory network analysis

To predict differentially expressed miRNAs (DEmiRs), we utilized the miRWalk database. The miRWalk database contains information about miRNA binding sites in various species, including humans, rats, cows, mice, and dogs (22). By analyzing the binding sites of known miRNAs to genes, we were able to identify potential target genes regulated by DEmiRs. To identify the target genes of DEmiRs and their overlap with the differentially expressed mRNAs (DEmRNAs), we compared the miRNA–target gene interactions with the gene expression data. This allowed us to extract the DEmRNAs that were targeted by DEmiRs, thereby exploring the potential regulatory relationship between miRNAs and mRNAs in the context of TOF.

To visualize and analyze the miRNA–target gene regulatory network, we employed Cytoscape software. Cytoscape allowed us to create a visual representation of the regulatory network, where miRNAs and their target genes were represented as nodes, and the interactions between them were depicted as edges. This network visualization aided in understanding the complex regulatory relationships between miRNAs and mRNAs involved in TOF.

### miRNA–mRNA–TF regulatory network analysis

To predict the TFs associated with ferroptosis-related differentially expressed mRNAs (DEmRNAs), we employed the DAVID (Database for Annotation, Visualization, and Integrated Discovery) Bioinformatics Resources. DAVID provides a comprehensive platform for functional annotation and enrichment analysis of gene lists. By inputting the list of DEmRNAs into DAVID, we were able to identify the potential TFs that may regulate the expression of these genes. DAVID utilizes various algorithms and databases to predict TF–gene interactions based on known binding motifs and regulatory relationships (23).

To visualize and explore the interactions between microRNAs, their potential targets, DEmRNAs, and their potential TFs, we used the Cytoscape software. Cytoscape allowed us to construct a visual regulatory network where microRNAs, their predicted targets, DEmRNAs, and predicted TFs were represented as nodes and the interactions between them were depicted as edges.

### Sample collection

We collected cardiac tissue samples from a cohort of 14 individuals, consisting of four patients diagnosed with TOF and four healthy controls. Additional details are presented in Table 1.

**Table 1:** Sample information.

	Age	Gender	Disease type	Application
1	7 months	Male	TOF	qPCR
2	5 months	Male	TOF	qPCR
3	1 year	Female	TOF	qPCR
4	1 year	Male	TOF	qPCR
5	37 years	Female	Induced labor	qPCR
6	33 years	Female	Induced labor	qPCR
7	29 years	Female	Induced labor	qPCR
8	29 years	Female	Induced labor	qPCR
9	7 months	Male	TOF	Immunofluorescence
10	6 months	Female	TOF	Immunofluorescence
11	1 year	Male	TOF	Immunofluorescence
12	25 years	Female	Induced labor	Immunofluorescence
13	35 years	Female	Induced labor	Immunofluorescence
14	30 years	Female	Induced labor	Immunofluorescence

### qPCR analysis

Total RNA was extracted from the tissue samples using TRIzol reagent (Takara, Dalian, China), which is a commonly used method for RNA extraction. The extracted RNA was then reverse transcribed into complementary DNA (cDNA) using the SYBR PrimeScript PLUS RT-PCR kit (Yeasen, Shanghai, China). This kit facilitates the conversion of RNA into cDNA and subsequent amplification of specific target sequences.

To design the primers required for PCR amplification, we used the Premier 5 primer design software. Specifically, we designed primers for the genes GAPDH, KEAP1, NFE2L2, FTH1, and SQSTM1. These primers are listed in Table 2. To detect and quantify the cDNA samples, we employed the SYBR PCR Master Mix kit (Vazyme, Nanjing, China). This kit enables the amplification of target sequences and quantification of the amplified products using real-time fluorescence PCR. Each cDNA sample was subjected to three parallel tests to ensure accuracy and reproducibility. For the PCR reaction, we utilized a 7500 Real-time fluorescence quantitative PCR system. This system allows for the amplification and detection of PCR products in real time by monitoring the fluorescence emitted during the reaction (Table 3).

**Table 2:** qPCR primers.

Primer	Sequence (5′>3’)	Length (bp)	Tm (°C)
KEAP1-F	GGTATGAGCCAGAGCGGATG	21	62.64
KEAP1-R	CGGCATAAAGGAGACGATTGAGGA	24	62.53
SQSTM1-F	GCAATGGGCCTGTGGTAG	18	58.08
SQSTM1-R	CCCGAAGTGTCCGTGTTT	18	57.55
NFE2L2-F	AGCGACGGAAAGAGTATGA	19	55.87
NFE2L2-R	GGGCAACCTGGGAGTAG	17	56.04
FTH1-F	CGCCTCCTACGTTTACC	17	54.23
MYB-F	GATGGGTTTTGCTCAGGC	18	56.36
MYB-R	GTAACGCTACAGGGTATGGA	20	56.45
EN1-F	TTCCAGGCAAACCGCTACATC	21	60.95
EN1-R	ACTCGCTCTCGTCTTTGTCCT	21	61.15
PAX3-F	TGCCGTCAGTGAGTTCCAT	19	58.94
PAX3-R	GCTTTCCTCTGCCTCCTTC	19	57.83
XBP1-F	ATGGATTCTGGCGGTATTG	19	55.01
XBP1-R	TGGGTAGACCTCTGGGAGC	19	60.00
ZNF423-F	TTTACACCTGCGATCACTGTC	21	58.32
ZNF423-R	GTTGTGGGTCGTCATCACCA	20	60.25
FTH1-R	TTCTCAGCATGTTCCCTC	18	53.82
GAPDH-F	GGAGCGAGATCCCTCCAAAAT	21	59.86
GAPDH-R	GGCTGTTGTCATACTTCTCATGG	23	59.38

**Table 3:** qPCR program.

Stage 1	Rep: 1	95°C	5 min
Stage 2	Reps: 40	95°C	10 s
		60°C	30 s
Stage 3	Rep: 1	95°C	15 s
		60°C	60 s
		95°C	15 s

To standardize the expression levels of the target genes, we normalized the data to the endogenous expression of GAPDH, which is a commonly used housekeeping gene. The relative mRNA expression levels were calculated using the equation 2^ - (ΔΔCT) (CT, circulating threshold; ΔΔCT = ΔCT_treated_ - ΔCT_control_, ΔCT = CT_gene_ − CT_housekeeping gene_).

### Histological analysis

Samples of the right ventricular tissue were fixed with 4% paraformaldehyde at 4°C overnight. After several washes with Phosphate Buffered Saline (PBS), the samples were cryopreserved in 30% sucrose and then embedded in O.C.T. (Tissue-Tek) stored at −80°C. Prior to immunofluorescence analysis, the tissue blocks were sectioned at a thickness of 8 µm using a cryostat (Leica CM3050 S).

The sections were allowed to come to room temperature for 30 min. After washing with PBS, the sections were blocked in a solution of PBS containing 5% goat serum for 1 h at room temperature. Then, the sections were incubated with the primary antibody (ab125066, 1:100) in a solution of PBS containing 1% goat serum overnight at 4°C. After bringing the sections to room temperature for another 30 min and washing with PBS, the sections were incubated with the secondary antibody (ab314333, 1:200) for 1 h at room temperature. Finally, the slides were mounted with Vectashield medium (Vector Laboratories) and DAPI before imaging.

The immunofluorescence images were obtained using Leica DM IL and analyzed with ImageJ (version 1.53a, NIH).

For the quantification of glutathione peroxidase 4 (GPX4) + areas in pixels, ImageJ was used to measure fluorescence intensity in the region of the right ventricle, and the values were normalized to background. Statistical analyses were performed using two-tailed Student's *t*-tests, assuming normality when the test was passed. The statistical values are presented as mean ± standard error of the mean (SEM). The following nomenclature was used to represent the results: ns (not significant), *p < 0.05, **p < 0.01, ***p < 0.001, and ****p < 0.0001.

## Results

### Differential expression of ferroptosis-related DEmRNAs

**Figure 1:**
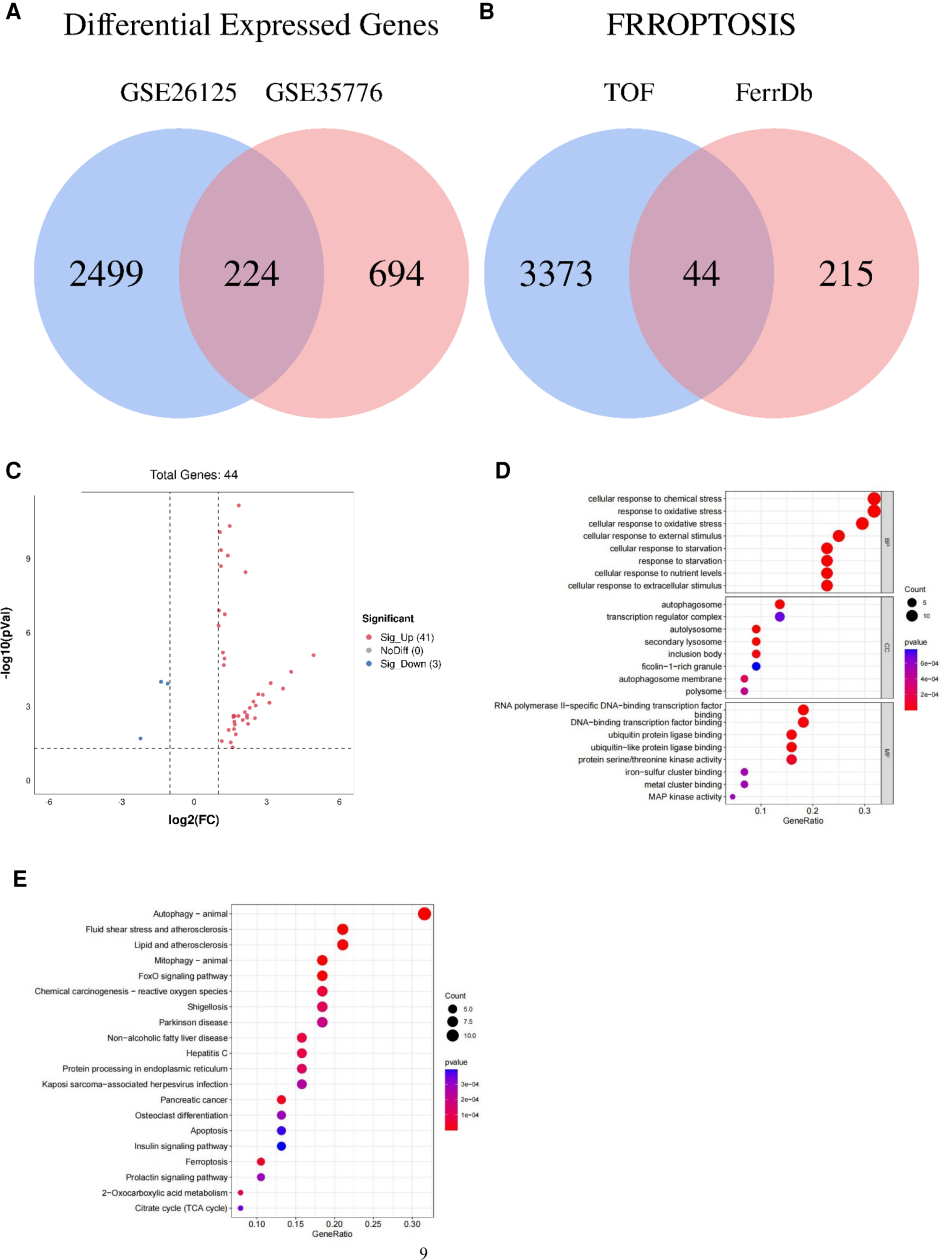
Venn diagram, volcano plot, and functional enrichment analyses of ferroptosis-related genes involved in tetralogy of Fallot. (**A**) Differentially expressed genes in TOF in the datasets of GSE26125 and GSE35776 were shown in the Venn diagram. (**B**) Differentially expressed genes concerned with ferroptosis in the datasets of GSE26125 and GSE35776 were shown in the Venn diagram. (**C**) Forty-four differentially expressed genes of ferroptosis in TOF were shown in the volcano plot: red represents the significantly upregulated genes, and the blue represents the significantly downregulated genes. (**D**) The top 24 GO analysis of differentially expressed genes related to ferroptosis in TOF. (**E**) The top 20 KEGG pathway analysis of differentially expressed genes related to ferroptosis in TOF.

Following quality control and data normalization processes, we conducted differential gene expression analysis and identified a total of 3,417 differentially expressed mRNAs (DEmRNAs). These DEmRNAs were obtained by combining the differentially expressed genes from the GSE35776 and GSE26125 datasets, as illustrated in Figure 1A.

To specifically identify the DEmRNAs associated with ferroptosis, we obtained a dataset of 259 genes related to ferroptosis from the FerrDb (ferroptosis database). We then compared this dataset with the GSE26125 and GSE35776 datasets to identify overlapping genes. The Venn diagram in Figure 1B and the volcano plot in Figure 1C depict the intersection of the DEmRNAs and the ferroptosis-associated genes.

This intersection of DEmRNAs and ferroptosis-associated genes consisted of a total of 44 genes, including 16 drivers, 14 suppressors, and 20 markers. These genes represent potential key players in the regulation of ferroptosis. For a detailed list of these genes, please refer to Table 4.

**Table 4:** Classification of ferroptosis-related genes in TOF.

	Driver	Suppressor	Marker
Up	ACO1, CS, GABARAPL1, GABARAPL2, GOT1, HMGB1, KEAP1, LPCAT3, MAPK1, MAPK9, NCOA4, PHKG2, PIK3CA, PRKAA2, RPL8, SLC38A1	AKR1C3, CISD1, FH, FTH1, HSPB1, ISCU, LAMP2, NFE2L2, PRDX6, RB1, SLC40A1, SQSTM1	ARRDC3, BNIP3, EIF2AK4, EIF2S1, FTH1, HERPUD1, HMGB1, HSPB1, MT3, NFE2L2, PRDX1, RELA, RPL8, SLC2A12, SLC40A1, STMN1, UBC, VLDLR, XBP1
Down		ARNTL, PROM2	DUSP1

### GO and KEGG pathway enrichment analysis of ferroptosis-related DEmRNAs

To gain a deeper understanding of the potential pathogenesis associated with the DEmRNAs involved in ferroptosis, we conducted functional and pathway enrichment analyses. These analyses provide insights into the biological processes and molecular pathways that may be impacted by the dysregulation of these genes.

For the functional enrichment analysis, we identified the significantly enriched GO terms within the ferroptosis-related DEmRNAs. The top eight GO terms included cellular response to chemical stress, cellular response to oxidative stress, response to oxidative stress, cellular response to starvation, response to starvation, cellular response to nutrient levels, cellular response to external stimulus, and cellular response to extracellular stimulus, as illustrated in Figure 1D.

In addition to functional enrichment, we also performed pathway enrichment analysis using the KEGG database. The top eight significantly enriched KEGG pathways associated with ferroptosis-related DEmRNAs were autophagy (animal), mitophagy (animal), fluid shear stress and atherosclerosis, FoxO signaling pathway, lipid and atherosclerosis, pancreatic cancer, ferroptosis, and chemical carcinogenesis (reactive oxygen species), as depicted in Figure 1E.

### PPI network construction of ferroptosis-related DEmRNAs

To further explore the relationship between the ferroptosis-associated DEmRNAs and TOF, we constructed a PPI network using the Cytoscape software. The PPI network was built based on the STRING database, which provides information on PPI. In addition, we utilized Cytoscape's application program CytoNCA to analyze the gene networks within the PPI network. Through this analysis, we identified several hub genes that play crucial roles in the regulation of ferroptosis-associated DEmRNAs involved in TOF.

**Figure 2:**
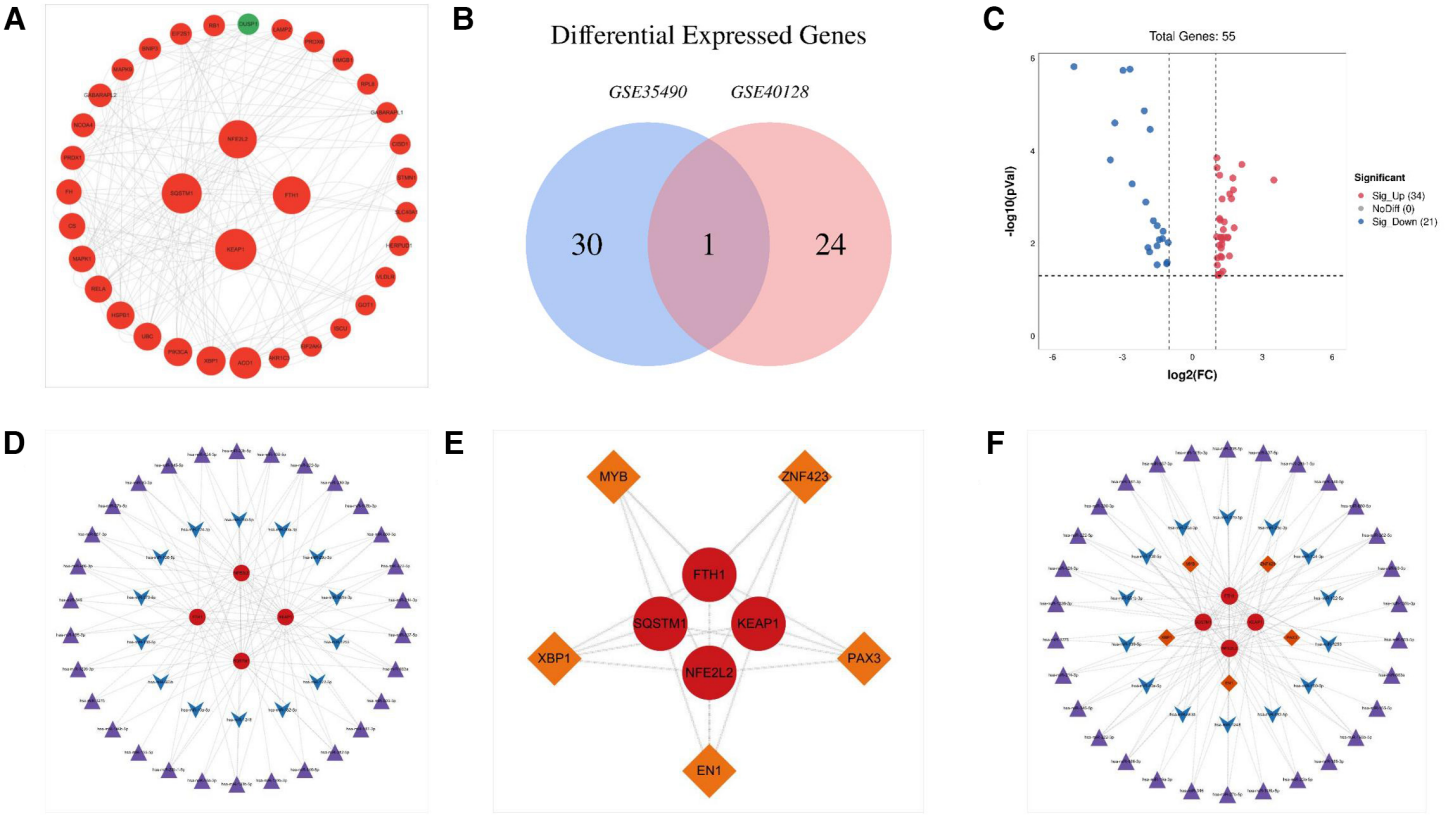
The PPI network of genes related to ferroptosis in tetralogy of Fallot and miRNA–mRNA–TF regulatory network of hub genes involved in tetralogy of Fallot. (**A**) The PPI network of ferroptosis-related genes involved in tetralogy of Fallot. (**B**) Differentially expressed microRNAs in TOF in the datasets of GSE35490 and GSE40128 were shown in the Venn diagram. (**C**) Fifty-five differentially expressed microRNAs of ferroptosis in TOF were shown in the volcano plot: red represents the significantly upregulated genes, and the blue represents the significantly downregulated genes. (**D**) miRNA–target regulatory networks of hub genes involved in tetralogy of Fallot. (**E**) The TF–target regulatory network of hub genes involved in tetralogy of Fallot. (**F**) miRNA–mRNA–TF regulatory network of hub genes involved in tetralogy of Fallot. Red circles manifest upregulated genes, and green circles manifest downregulated genes. The four larger circles manifest hub genes, and the smaller circles manifest non-hub genes. Purple triangles manifest upregulated miRNAs, and blue arrows manifest downregulated miRNAs. Orange diamonds manifest TFs. TFs, transcription factors.

Specifically, we found that the genes KEAP1, SQSTM1, NFE2L2, and FTH1 had high betweenness centrality within the gene network. These hub genes are significantly upregulated in TOF, as depicted in Figure 2A. The high betweenness centrality suggests that these genes act as key mediators or connectors within the network, potentially playing important roles in regulating ferroptosis in TOF.

### miRNA–TF–target regulatory network analysis of ferroptosis-related DEmRNAs

To investigate the regulatory relationship between DEmiRs and differentially expressed mRNAs (DEmRNAs) in TOF, we downloaded the datasets GSE35490 and GSE40128 from the GEO database. Through analysis, we identified a total of 55 DEmiRs, of which 21 were downregulated DEmiRs and 34 were upregulated DEmiRs (Figures 2B,C). To predict the target genes of these DEmiRs, we utilized miRWalk software. We then compared the predicted target genes with the ferroptosis-related DEmRNAs, resulting in the construction of a miRNA–target network (Figure 2D). This network provides insight into the potential regulatory relationships between the DEmiRs and DEmRNAs involved in ferroptosis in TOF.

Furthermore, we predicted that several TFs may regulate the expression of the ferroptosis-associated DEmRNAs in TOF. The predicted TFs included MYB, EN1, PAX3, XBP1, and ZNF423. To determine the regulatory connections between these TFs and their target DEmRNAs, we constructed a TF–target regulatory network. This network consisted of nine nodes and 20 pairs of interactions, wherein MYB, EN1, PAX3, XBP1, and ZNF423 targeted FTH1, KEAP1, NFE2L2, and SQSTM1 (Figure 2E).

Finally, to comprehensively understand the regulatory network involving DEmiRs, DEmRNAs, and TFs, we constructed a microRNA–mRNA–TF regulatory network. This network incorporated 44 DEmiRs, four DEmRNAs, and five TFs, and it provides a holistic view of the potential regulatory interactions involved in ferroptosis in TOF (Figure 2F).

### Real-time fluorescence quantitative PCR detection of expression levels of hub genes and TFs

**Figure 3:**
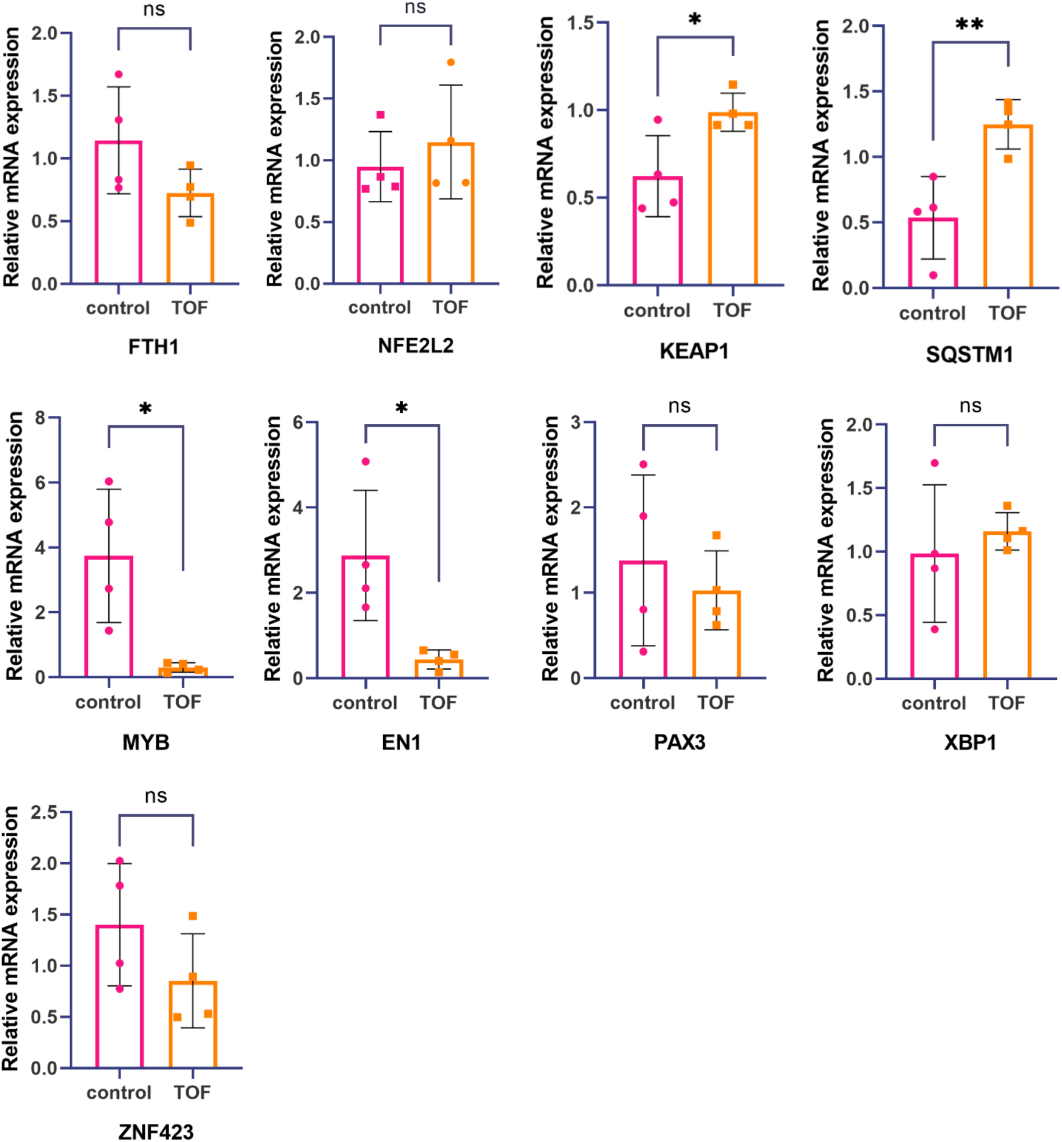
Hub gene and TF's expression levels were measured by real-time fluorescence quantitative PCR. The mRNA expression levels of KEAP1 and SQSTM1 in relation to tetralogy of Fallot were significantly higher compared with the healthy control group. On the other hand, the mRNA expression levels of MYB and EN1 related to tetralogy of Fallot were significantly lower than those in the healthy control group. However, no significant differences were observed in the mRNA expression levels of FTH1, NFE2L2, PAX3, XBP1, and ZNF423 between tetralogy of Fallot patients and the healthy control group. Statistical significance is shown as ∗*p* < 0.05 and ∗∗*p* < 0.01.

To confirm the role of hub genes (FTH1, NFE2L2, KEAP1, and SQSTM1) and TFs (EN1, MYB, PAX3, ZNF423, and XBP1) in ferroptosis associated with TOF, real-time fluorescence quantitative PCR analysis was conducted. The expression levels of these genes were compared between TOF samples and the control group. The results showed a significant upregulation of KEAP1 and SQSTM1 in the TOF samples, suggesting their potential involvement in the dysregulation of ferroptosis in TOF. In addition, MYB and EN1 were found to be significantly downregulated in the TOF samples. This suggests that the decrease in MYB and EN1 may be responsible for the upregulation of KEAP1 and SQSTM1, thus contributing to ferroptosis in TOF. However, no significant difference was observed in the expression levels of FTH1, NFE2L2, PAX3, XBP1, and ZNF423 between the TOF samples and the control group (Figure 3).

### Immunofluorescence analysis of ferroptosis

**Figure 4:**
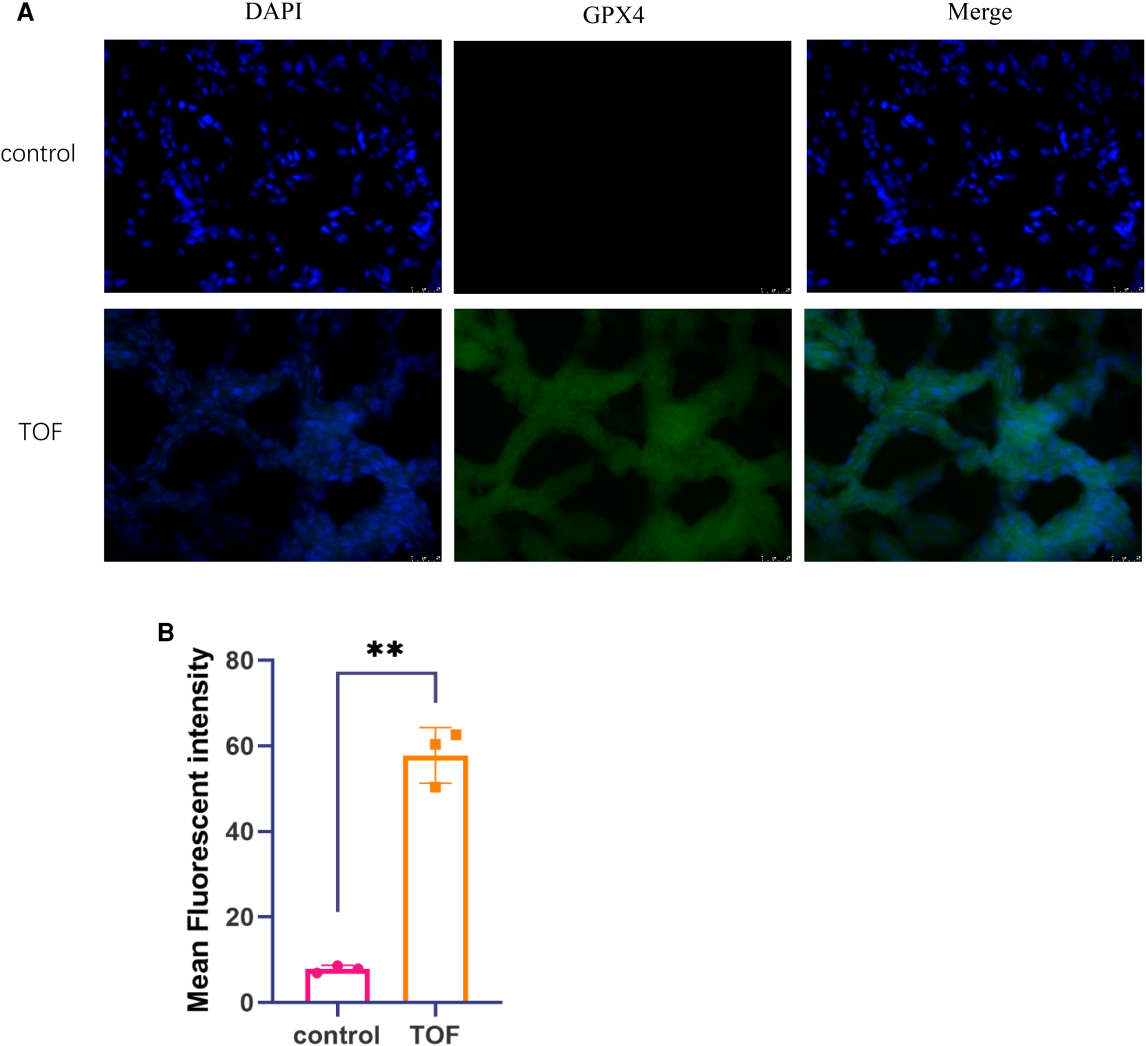
The levels of ferroptosis were assessed through immunofluorescence analysis. (**A**) Representative immunofluorescence images of TOF samples (*n* = 3) and control samples (*n* = 3) are shown. The scale bar indicates 25 μm. DAPI fluorescence is depicted in blue, GPX4 fluorescence is represented in green, and the merged image is shown in cyan. (**B**) The mean fluorescent intensity of TOF samples (*n* = 3) and control samples (*n* = 3) is presented. Statistical significance is indicated as ∗*p* < 0.05 and ∗∗*p* < 0.01.

To examine the levels of ferroptosis in both TOF and control groups, we performed immunofluorescence and ImageJ analysis. Our results revealed a significant increase in GPX4 levels in TOF samples compared with the control samples (Figure 4).

## Discussion

Abnormalities in the cardiac OFT are among the most prevalent congenital heart diseases, affecting approximately 22.7% of individuals. These abnormalities may be indicative of intricate morphogenetic events that occur during fetal heart development (24). The proper development of the conotruncus, the embryonic precursor of the OFT, is crucial for normal cardiac development. The separation of the OFT contributes to the formation of the aortic and pulmonary trunks and requires the coordinated interaction of various cell types, namely, second cardiac field cells, endocardial cells, epicardial cells, and cardiac neural crest cells. During this process, the myocardium in the OFT undergoes twisting before and during the separation phase. Any disruption or imbalance in OFT separation can lead to the development of TOF. TOF is a complex congenital heart defect characterized by the combination of multiple malformations, such as a ventricular septal defect, overriding aorta, pulmonary stenosis, and right ventricular hypertrophy. The underlying pathogenesis of TOF involves abnormalities in OFT development, particularly in the separation and alignment of the aorta and pulmonary artery. Such developmental defects can ultimately result in the clinical manifestations observed in TOF (25, 26).

Recent studies have demonstrated that ferroptosis plays a pivotal role in embryonic development. The occurrence of congenital heart diseases is closely linked to ferroptosis, possibly due to elevated levels of iron ions in cardiac tissues. These high iron levels can trigger lipid peroxidation through catalytic oxidation reactions, leading to cellular membrane damage and subsequent cell death. Such cell deaths may contribute to the development of cardiac malformations and cardiovascular diseases. Various regulatory mechanisms of ferroptosis, such as the actions of lipid bilayer radicals, lipid peroxidation reactions, and lipid peroxide metabolism, are closely intertwined with the onset and progression of heart diseases (27). In the context of human embryonic stem cells (hESCs), which possess the remarkable capacity to differentiate into nearly all types of somatic cells *in vitro*, the process of cellular separation can be prone to cell death. Research has indicated that iron accumulation and subsequent lipid peroxidation are underlying contributors to dissociation-induced cell death in hESCs. Furthermore, it has been observed that the reduction in the activity of GPX4 caused by iron accumulation promotes the occurrence of ferroptosis (28).

In order to gain further insight into the potential involvement of ferroptosis in the pathogenesis of TOF, we conducted analyses using GEO and FerrDb datasets to identify differentially expressed mRNAs and microRNAs related to ferroptosis in TOF. Our analysis revealed 41 upregulated genes and seven downregulated genes that are implicated in ferroptosis in TOF. Subsequently, we performed GO enrichment analyses to explore the functional roles of these genes. The results indicated that these genes are primarily involved in cellular response to chemical stress, cellular response to oxidative stress, response to oxidative stress, cellular response to starvation, response to starvation, cellular response to nutrient levels, and cellular response to external and extracellular stimuli. Furthermore, we conducted KEGG pathway analysis to elucidate the potential molecular mechanisms underlying TOF. The pathways identified were autophagy (animal), mitophagy (animal), fluid shear stress and atherosclerosis, FoxO signaling pathway, lipid and atherosclerosis, pancreatic cancer, ferroptosis, and chemical carcinogenesis (reactive oxygen species). These pathways shed light on the molecular mechanisms that may be involved in TOF.

During the developmental process of the heart, oxidative stress can cause damage to proteins, DNA, and lipids, thereby affecting the growth, repair, and maintenance of cardiac tissues. In the formation of congenital heart defects, oxidative stress can disrupt the structure and function of the early embryonic heart tube, leading to abnormal heart development. Consequently, reducing the degree of oxidative stress can minimize the impact on fetal heart development, thereby helping to prevent and decrease the occurrence of congenital heart defects (29). Furthermore, the inhibition of lamprey immune protein (LIP) can impede development, resulting in embryo death, abnormal morphology in various tissues, and severe growth retardation. This mechanism may be attributed to the overexpression of LIP triggering ferroptosis, which leads to the accumulation of ROS and oxidative stress damage. Consequently, this can lead to cell death and pericardial edema (30). Hence, it can be deduced that ferroptosis plays a role in the development of TOF by modulating oxidative stress responses. Disrupted iron metabolism leads to heightened oxidative stress, subsequently triggering lipid peroxidation and cellular damage, ultimately impacting the proper development of the embryonic heart.

KEAP1 is a crucial protein involved in regulating cellular oxidative stress responses, alongside NRF2. When cells experience oxidative stress, KEAP1 binds to NFE2L2/NRF2 and modulates its stability through conformational changes at Cys62. This interaction leads to the dissociation of NFE2L2/NRF2 from KEAP1 and its translocation into the nucleus, where it acts as a TF to regulate the expression of various antioxidant and detoxification genes. The upregulation of these genes enhances the cell's antioxidant and detoxification capabilities, thus safeguarding it against oxidative stress-induced damage. In addition, KEAP1 can also inhibit NFE2L2/NRF2 expression (31, 32). Increased levels of ROS alter the expression of specific genes vital for the differentiation of cardiac neural crest cells, thereby leading to cardiac defects during embryonic development. In zebrafish, there are two NFE2L2/NRF2 genes, NFE2L2/NRF2a and NFE2L2/NRF2b, with the latter exhibiting negative regulation during development (33).

Recent studies have indicated that SQSTM1/p62 plays a role in the autophagic degradation of ARNTL specifically in type 2 ferroptosis inducers, rather than type 1 ferroptosis inducers (34). In addition, SQSTM1/p62 has been shown to inhibit ferroptosis by activating the NRF2 signaling pathway (35). It has been observed that the disruption of the interaction between NCOA4 and FTH1 can inhibit ferroptosis (36, 37). SQSTM1/p62 primarily plays a role in heart diseases through autophagy, and currently, there is insufficient evidence to support the role of SQSTM1/p62 in congenital heart diseases through ferroptosis.

FTH1 and ferritin light chain (FTL) genes encode the two subunits of the widely distributed intracellular ferritin complex, which functions as a controlled iron storage and release system. FTH1 possesses ferroxidase activity and is responsible for the conversion of ferrous iron (Fe2+) to ferric iron (Fe3+), while FTL primarily facilitates iron nucleation and stabilizes assembled ferritin proteins (38). In recent studies, the NAMPT-Sirt1-FOXO1-FTH1 signaling axis has emerged as a crucial player in regulating cardiomyocyte ferroptosis and safeguarding heart function against the detrimental effects of ischemia/reperfusion (I/R) injury. This signaling pathway acts as a protective mechanism, contributing to the preservation of cardiac health during I/R injury (39). However, the specific role of FTH1 in congenital heart diseases through the ferroptosis pathway is still not fully understood, and further research is needed to unravel its involvement in these conditions.

The importance of microRNAs in normal heart development is evident from studies involving specific knockout of Dicer, an RNase required for microRNA processing and biosynthesis, in mouse tissues. In the vascular lineage, it has been observed that the loss of Dicer leads to a lethal phenotype between the 16th and 17th day of embryo development that is characterized by extensive internal bleeding caused by thin-walled vessels (40). During its early embryonic development, alterations in microRNA expression can contribute to the development of congenital heart diseases, which are the most common birth defects in humans (41). In our study, we identified 14 downregulated microRNAs and 30 upregulated microRNAs that are predicted to be associated with four hub genes. Notably, has-miR-222 has been reported to regulate cardiac microRNA maturation and trigger the development of TOF (7).

Among the five TFs identified in our study, MYB stands out as it is unable to generate embryoid bodies (EBs) containing spontaneously contractile vascular smooth muscle cells. However, it is capable of differentiating into intact contractile cardiomyocytes (42). In addition, MYB has been found to play a vital role in regulating the proliferation of vascular smooth muscle cells (43).

PAX3 was initially detected during mouse embryonic development at E6.5, where it was found to be located in the dorsal part of the neuroepithelium and a subset of myomeres until E8.5. From E9.5 to E12.5, PAX3 expression becomes more widespread, such as in cardiac neural crest cells. However, by E17.5, PAX3 expression is no longer detected (44). Interestingly, studies have shown that hyperglycemia and oxidative stress induced on the 7.5th day of gestation in mice can disrupt the subsequent migration of cardiac neural crest cells and result in defects in the cardiac outflow tract. However, treating mice to normalize their oxidant status and prevent the inhibition of PAX3 expression can rescue this phenotype (45).

Activation of XBP1 splicing, induced by VEGF, has been identified as a critical process in angiogenesis (46). XBP1 exerts its influence through the growth factor signaling pathway, thereby regulating endothelial cell proliferation and promoting angiogenesis (47).

In light of these findings, we hypothesize that alterations in ferroptosis-related genes may contribute to the pathogenesis and progression of TOF, thereby offering novel avenues for understanding the underlying mechanisms of this condition. Nevertheless, it is important to acknowledge the limitations of our study. First, the availability of clinical TOF samples was limited, which hindered our ability to validate the expression patterns of all TFs in the tissues of the human right ventricle. Second, the levels of ferroptosis in the samples were not directly measured; therefore further investigation is required. These challenges highlight areas for future research in this field.

## Conclusion

Through bioinformatics analysis, we identified 44 potential ferroptosis-related genes in TOF. Among these, KEAP1 emerged as particularly noteworthy, suggesting their potential involvement in the development of the outflow tract by regulating ferroptosis. These findings have the potential to enhance our understanding of TOF pathogenesis and may contribute to improved diagnostic approaches for this condition. It is important to note that further experimental validation is needed to confirm the role of these genes in TOF.

## Data Availability

The datasets presented in this study can be found in online repositories. The names of the repository/repositories and accession number(s) can be found in the article/Supplementary Material.
